# UAV remote sensing for yield prediction in staple crops: a review

**DOI:** 10.3389/fpls.2026.1840669

**Published:** 2026-07-14

**Authors:** Peihan Zhao, Wenteng Li, Chao Wang, Bin Wang, Tao Yang, Xinxin Wang, Zhen Liu, Hongquan Liu, Fushun Wang

**Affiliations:** 1 College of Information Science and Technology, Hebei Agricultural University, Baoding, China; 2 Hebei Key Laboratory of Agricultural Big Data, Baoding, China; 3 State Key Laboratory of Crop Gene Resources and Breeding/Institute of Crop Sciences, Chinese Academy of Agricultural Sciences, Beijing, China; 4 College of Horticulture, Hebei Agricultural University, Baoding, China; 5 Cangzhou Academy of Agriculture and Forestry Sciences, Cangzhou, China; 6 College of Urban and Rural Construction, Hebei Agricultural University, Baoding, China; 7 State Key Laboratory of North China Crop Improvement and Regulation, Baoding, China; 8 Key Laboratory of Water-saving Agriculture in North China, Ministry of Agriculture and Rural Affairs, Baoding, China

**Keywords:** crop yield prediction, ground-truth yield, spatial scale, spatiotemporal modeling, UAV remote sensing

## Abstract

Accurate yield prediction for major grain and oilseed crops, including soybean, corn, wheat, and rice, is essential for food-security assessment and precision field management. This study presents a structured integrative review of UAV-based crop yield prediction and follows PRISMA-guided procedures for literature search, screening, and evidence synthesis. Seventy peer-reviewed studies published between 2018 and 2025 were synthesized within a “Data–Ground Truth–Model–Decision” framework. Beyond summarizing UAV platforms, sensor configurations, feature-engineering strategies, and model architectures, the review explicitly distinguishes among microplot, field, and regional prediction scales, and evaluates the characteristics and limitations of yield-label acquisition methods, including manual harvest, plot-combine harvest, and combine yield-monitor data. Existing evidence indicates that the reliability of UAV-based yield prediction depends not only on optimal image acquisition windows, multi-source feature fusion, and model architecture, but also on scale-consistent yield labels, spatially aware validation strategies, and clearly defined model outputs, such as plot-level scalar yield, field-scale yield maps, and regional yield estimates. Major bottlenecks include scale mismatch between UAV imagery and yield labels, error propagation during yield-map generation, limited cross-year and cross-region transferability, weak causal interpretability, and difficulties in deploying models under complex operational field conditions. Future research should emphasize scale-explicit benchmark datasets, quality-controlled ground-truth yield acquisition, UAV–satellite–ground data fusion, spatiotemporal deep learning, and edge–cloud collaborative systems that can translate prediction outputs into agronomic decisions. This review provides a practical pathway for developing robust, interpretable, and deployable UAV-based yield prediction systems for major grain and oilseed crops.

## 1 Introduction

As the global population continues to grow and concerns about food security intensify (Kheir et al., 2021), enhancing the accuracy, timeliness, and precision of yield predictions for major crops has emerged as a critical challenge in advancing precision agriculture (Guebsi et al., 2024; Shafi et al., 2019).Traditional methods for yield prediction, which primarily depend on ground sampling and statistical models, are constrained by significant limitations in monitoring scope, data resolution, and the ability to perform spatiotemporal dynamic analysis. Recently, Unmanned Aerial Vehicle (UAV) technology has been rapidly adopted in agricultural remote sensing, valued for its high spatiotemporal resolution, cost-effectiveness, and flexible deployment (Ballesteros et al., 2018; Cen et al., 2019; Doughty and Cavanaugh, 2019). This has opened up a new technological pathway for predicting crop yields.

Equipped with a diverse array of sensors (Li et al., 2016; Lin et al., 2018; Yue et al., 2017), such as visible light, multispectral, thermal infrared, and hyperspectral cameras—UAVs are capable of acquiring extensive physiological and environmental data from crops at critical growth stages or across multiple time points (Kurihara et al., 2023; Li et al., 2022; Liu et al., 2023; Zhang et al., 2020).From the high-resolution spectral, thermal infrared, and RGB images collected, key information, including vegetation indices (Fei et al., 2022; Kurihara et al., 2023; Liu et al., 2022)and texture features (Skobalski et al., 2024), can be extracted to effectively characterize crop growth status, population structure, and canopy conditions (Jiang et al., 2024; Wan et al., 2020).Simultaneously, the emergence of machine learning and deep learning methods provides powerful tools for efficient feature extraction and yield prediction from the comprehensive data provided by UAV remote sensing, thereby further improving prediction accuracy (Muruganantham et al., 2022).

Although substantial research has investigated UAV remote sensing for yield prediction in major staple crops such as soybean, corn, wheat, and rice (da Silva et al., 2020; Fu et al., 2020; Furukawa et al., 2020), the central bottleneck in this field is no longer confined to sensor selection or algorithmic performance. Yield prediction is essentially a supervised spatial modeling problem; therefore, model outputs can be properly interpreted only when the spatial support of UAV-derived features, the method used to obtain ground-truth yield, and the intended prediction target are explicitly aligned. For example, a plot-level model trained using destructive harvest data cannot be assumed to generate reliable sub-field yield prescription maps, whereas a model trained using combine yield-monitor data must account for measurement lag, swath-width uncertainty, moisture correction, and geolocation errors (A Sudduth et al., 2012; Lyle et al., 2014). Accordingly, this review extends the original “Data–Model–Decision” perspective by incorporating two critical components that are often treated implicitly in the existing literature: spatial scale definition and ground-truth yield acquisition. This expanded framework provides a more comprehensive “Data–Ground Truth–Model–Decision” perspective for evaluating the reliability and practical applicability of UAV-based crop yield prediction.

From a global research perspective, studies on UAV-based crop yield prediction are rapidly expanding, with notable innovations in multimodal data fusion, temporal feature mining, and the deployment of lightweight models. However, research fragmentation and a lack of an engineering-oriented approach have resulted in many advanced models being confined to the experimental validation stage, preventing the establishment of reproducible and scalable technical standards. Consequently, there is a pressing need to systematically review the existing literature, delineate the primary trajectory of technological advancement, pinpoint key bottlenecks, and bridge the gap between research and practical application.

To address these issues, the present manuscript is positioned as a structured integrative review based on systematic search and transparent screening procedures. It synthesizes 70 representative peer-reviewed studies published between 2018 and 2025 and follows a revised conceptual pathway from data acquisition, ground-truth construction, feature processing, and model input–output mapping to model validation and decision translation. The year 2018 was selected as the starting point because, after 2017, low-cost UAV multispectral/RGB platforms, high-resolution field phenotyping workflows, and deep-learning techniques expanded rapidly in crop monitoring, shifting UAV-based yield prediction from isolated image acquisition toward multi-source data fusion and intelligent modeling. The crop scope is limited to soybean, corn, wheat, and rice because these four major grain crops collectively constitute the dominant evidence base in current UAV-based yield-prediction studies and represent contrasting agronomic application scenarios, including breeding microplots, commercial row-crop fields, and regional grain-production systems. Therefore, this review does not aim to enumerate all possible agricultural applications of UAVs. Instead, it critically evaluates how spatial scale, yield labels, feature representations, model architectures, and deployment constraints jointly determine whether UAV-based crop yield prediction can move beyond experimental validation toward field-ready decision support for agronomic management.

The literature search was restricted to studies published between 2018 and 2025 because this period captures a clear methodological transition in UAV-based crop monitoring, from isolated image acquisition and single vegetation-index regression toward high-resolution phenotyping, multi-source feature fusion, and machine-learning/deep-learning modeling supported by low-cost RGB/multispectral UAV platforms (Xie and Yang, 2020; Gano et al., 2024).The crop scope was limited to soybean, corn, wheat, and rice based on their relevance to global food, feed, and oilseed security and on the availability of a sufficiently developed UAV yield-prediction evidence base. Corn, wheat, and rice are the dominant global cereal crops, together accounting for 91% of total global cereal production in 2023 FAO (FAO Agricultural production statistics 2010–2023). Although soybean is not a cereal crop, it is a globally important oilseed and plant-protein crop with a central role in edible oil, feed protein, and oilseed value chains; soybean meal dominates global protein-meal production from oilseed crush and accounts for more than two-thirds of world protein-meal production OECD-FAO (OECD-FAO Agricultural Outlook 2025–2034, Oilseeds and oilseed products). Therefore, the term “major crops” in this review is operationally defined as representative major grain and oilseed crops with strong relevance to global food, feed, and oilseed systems, namely soybean, corn, wheat, and rice.

## 2 Review protocol

The present review was designed as a structured integrative review following PRISMA-guided procedures for literature search, screening, and reporting. Because the included studies varied substantially in crop species, UAV sensors, spatial support, ground-truth yield measurement, model inputs, output targets, and validation schemes, the evidence was synthesized through a structured integrative framework. This approach enabled heterogeneous methodological evidence to be organized around data acquisition, yield-label construction, feature processing, model design, validation, and decision-oriented application (Page et al., 2021; Snyder, 2019). The PICOS definition and review scope used in this structured integrative review are summarized in **Table 1**.

**Table 1:** PICOS definition and review scope used in this structured integrative review.

PICOS element	Definition used in this review	Operational implication for screening and synthesis
Population/Problem	Field-based yield prediction for soybean, corn, wheat, and rice under UAV remote sensing scenarios.	Restricts the review to major grain and oilseed crops with published UAV yield-prediction evidence.
Intervention/Index method	UAV-derived RGB, multispectral, hyperspectral, thermal, LiDAR, structural, texture, temporal, or fused features, with or without ancillary weather/soil data.	Focuses on UAV-enabled data streams rather than satellite-only or ground-sampling-only studies.
Comparator/Context	Differences among sensor types, flight periods, feature sets, model families, spatial scales, and validation strategies.	Supports critical comparison rather than a single pooled effect size.
Outcomes	Yield prediction accuracy (R², RMSE, RRMSE/NRMSE), output form (plot scalar, field map, regional estimate), transferability, interpretability, and deployability.	Requires each study to report a measurable yield-prediction output or sufficient methodological detail.
Study design	Peer-reviewed empirical studies and peer-reviewed conference or methodological studies that provided sufficient information on UAV data, ground-truth yield acquisition, model construction, validation strategy, and yield-prediction output.	Excludes satellite-only, non-peer-reviewed, inaccessible, or methodologically insufficient records.

### 2.1 Search strategy

The literature search was conducted using Google Scholar and Web of Science as the core retrieval platforms. The final search was completed on June 28, 2025. Searches were limited to English-language peer-reviewed studies published between 2018 and 2025. In Web of Science, the search strings were applied to topic fields, including title, abstract, author keywords, and Keywords Plus. In Google Scholar, the first 200 results for each core and supplementary search string were screened, and records were retained only when the title, abstract, or full text was directly related to UAV-based yield prediction in soybean, corn, wheat, or rice. This period was selected to capture the transition of UAV-based crop yield prediction from single-index regression toward multi-source feature fusion and machine-learning/deep-learning modeling. PRISMA-guided procedures were used to improve the transparency of record identification and study selection. The structured integrative-review approach was adopted to synthesize heterogeneous methodological evidence across data acquisition, yield-label construction, feature processing, model design, validation, and decision-oriented application (Page et al., 2021; Snyder, 2019).

The core query was as follows: (“UAV” OR “drone” OR “unmanned aerial vehicle” OR “UAS”) AND (“crop yield prediction” OR “yield estimation” OR “grain yield prediction” OR “yield mapping”) AND (“soybean” OR “maize” OR “corn” OR “wheat” OR “rice”). To minimize potential bias arising from database coverage and terminology variation, supplementary searches were performed by combining sensor- and model-related terms with crop- and yield-related terms, including (“multispectral” OR “hyperspectral” OR “RGB” OR “LiDAR” OR “thermal”) AND (“machine learning” OR “deep learning” OR “CNN” OR “random forest”) AND (“yield”). In addition, backward and forward citation chasing was conducted to identify relevant studies that were not retrieved by the core search strings but met the predefined inclusion criteria. This strategy was used to improve the completeness of the literature retrieval, reduce terminology-related retrieval bias, and minimize the risk of missing eligible peer-reviewed studies.

### 2.2 Selection process

The study selection process consisted of preliminary screening, full-text assessment, and citation chasing. During preliminary screening, titles, abstracts, and keywords were examined to exclude records that were clearly unrelated to UAV-based crop yield prediction or lacked substantial methodological content. The remaining studies were then assessed through full-text reading. Studies that did not meet the inclusion criteria, lacked sufficient information on UAV data, yield labels, model construction, or validation, or did not provide a measurable yield-prediction output were excluded. During citation chasing, the reference lists of eligible and highly relevant studies were examined, and targeted searches were conducted in Google Scholar to identify additional studies that may have been missed by the database search.

Full-text screening and evidence coding were conducted using a predefined methodological adequacy checklist and a structured data-extraction form tailored to UAV-based crop yield prediction, with emphasis on whether each study clearly reported UAV data sources, ground-truth yield acquisition, model inputs, output targets, validation strategy, accuracy metrics, and major limitations. For each eligible study, the following information was extracted and coded: crop species, spatial scale, UAV platform, sensor type, image acquisition stage, preprocessing workflow, ground-truth yield source, feature type, model family, output target, validation strategy, accuracy metrics, and limitation reporting. This coding structure was used to ensure that the synthesis did not remain a simple inventory of algorithms, but instead linked model performance to data source, yield-label quality, spatial scale, validation reliability, and intended application target. The inclusion and exclusion criteria used during full-text assessment are summarized in **Table 2**. Grey literature, including dissertations, commercial reports, technical reports, and non-peer-reviewed online materials, was excluded to improve methodological comparability and source reliability. This restriction may reduce heterogeneity but may also introduce publication bias, which was considered when interpreting the evidence base.

**Table 2:** Study selection criteria used for literature screening.

Criterion type	Criterion used in this review	Screening implication
Inclusion	The study focused on UAV-based crop yield prediction or yield estimation.	Ensures direct relevance to the review topic.
Inclusion	The crop included soybean, corn, wheat, or rice.	Matches the defined crop scope of this review.
Inclusion	UAV-derived data were used as a major input source.	Excludes satellite-only or ground-sampling-only studies.
Inclusion	The study reported a measurable yield-prediction output or provided sufficient methodological detail for interpretation.	Ensures that model performance and workflow information can be extracted.
Inclusion	The study was published in a peer-reviewed journal or peer-reviewed conference proceeding.	Improves the reliability and comparability of the included evidence.
Exclusion	The full text was inaccessible.	Prevents incomplete evidence extraction.
Exclusion	The study lacked key information on UAV data, yield labels, model construction, or validation strategy.	Excludes methodologically insufficient studies.
Exclusion	The study focused only on crop monitoring, disease detection, biomass estimation, or phenotyping without yield prediction.	Maintains the focus on yield-prediction studies.
Exclusion	The study used only satellite or ground-sampling data without UAV-derived inputs.	Maintains the UAV-based review scope.
Exclusion	The study was a non-peer-reviewed blog, commercial report, technical note, or other grey-literature source outside the defined review scope.	Reduces uncertainty from non-peer-reviewed evidence.

### 2.3 Literature synthesis

**Figure 1:**
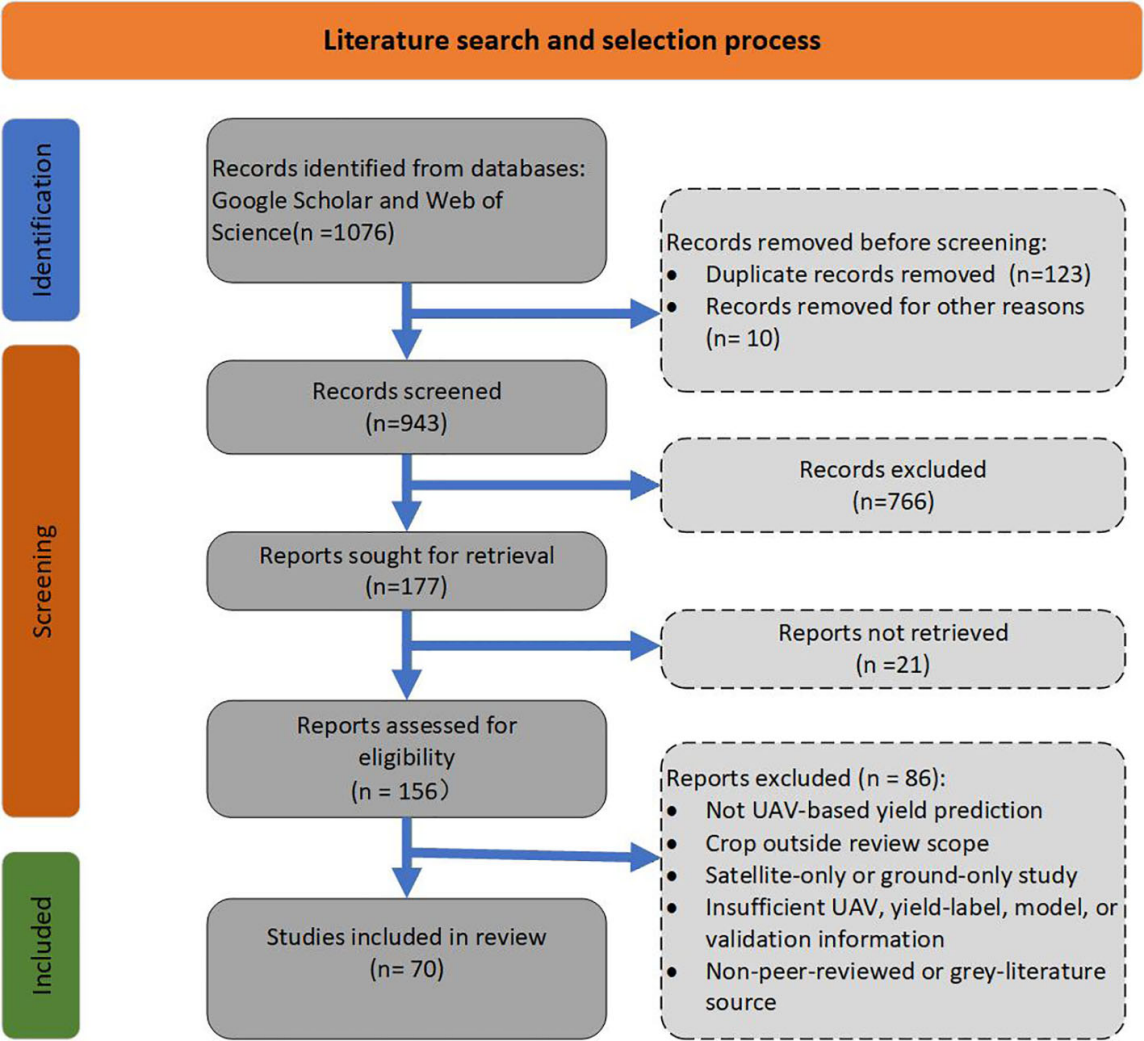
Literature search and selection process. Literature search and selection process. A total of 1, 076 records were identified from Google Scholar and Web of Science. After removing duplicate records and records excluded for other reasons, 943 records were screened. A total of 177 reports were sought for retrieval, of which 21 were not retrieved. The remaining 156 full-text reports were assessed for eligibility, and 86 reports were excluded because they were not focused on UAV-based yield prediction, involved crops outside the review scope, used satellite-only or ground-only data, lacked sufficient information on UAV data, yield-label acquisition, model construction, or validation strategy, or were non-peer-reviewed or grey-literature sources. Finally, 70 studies were included in the review.

Using the search and screening strategy described above (**Figure 1**), 70 peer-reviewed studies were retained for detailed synthesis. Because these studies differed substantially in crop species, sensor configurations, spatial support, yield-label sources, model structures, and validation designs, the evidence was not statistically pooled. Instead, a structured synthesis was conducted around five key dimensions: spatial scale, ground-truth yield acquisition, data processing, model input–output mapping, and field-deployment bottlenecks. This approach was intended to move beyond a purely descriptive inventory of previous studies and to provide a critical, application-oriented interpretation of the evidence.

**Figure 2:**
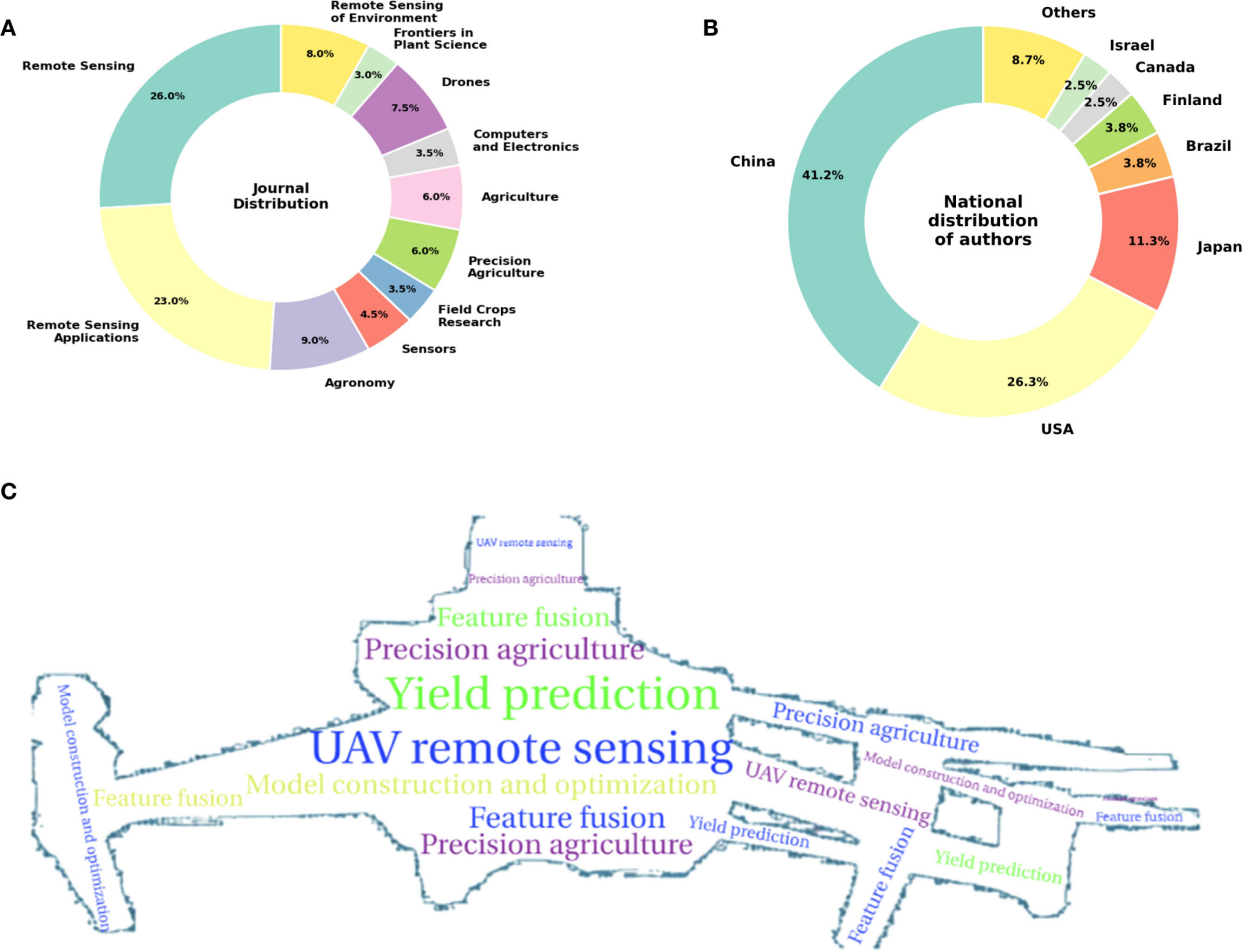
Bibliometric Visualization of UAV Remote Sensing for Crop Yield Prediction Research. **(A)** Journal Distribution; **(B)** Author Country Distribution; **(C)** Abstract Keyword Word Cloud.

As shown in **Figure 2**, a concentrated attention on this topic is evident among core journals in the field of remote sensing. Since remote sensing technology is an important supporting technology for UAV crop yield prediction, such journals are highly specialized and have significant influence. They are therefore more likely to publish and disseminate studies on UAV-based crop yield prediction.

From the perspective of the countries where the authors are from, China (41.2%) and the United States (26.3%) dominate. This is primarily attributed to substantial research investments and policy support in agricultural remote sensing, UAV technology, and artificial intelligence in both countries, Moreover, the two countries have extensive agricultural application scenarios, which has led to more abundant research outputs in this area. Furthermore, the keyword word cloud in the abstract further indicates that the current core focus of the research is on Yield, Prediction, Crop, and UAV, suggesting that the integration of machine learning, deep learning, and UAV remote sensing technology for crop yield prediction has become a research hotspot in this field.

## 3 Literature analysis

### 3.1 Overview

After completion of the literature search and selection process, the selected studies were found to share a broadly similar technical chain but to differ substantially in spatial support and prediction targets. Therefore, UAV-based crop yield prediction should not be interpreted merely as a simple regression from image-derived features to yield values. Rather, it should be understood as a supervised modeling process that maps UAV observations to ground-truth yield labels within a defined spatial scale. Based on this understanding, the revised conceptual framework consists of five interlinked components: UAV data acquisition, image preprocessing and feature extraction, yield-label construction, model input–output mapping, and decision-level application. This scale-aware framework is used as the organizing basis for the subsequent analysis to avoid conflating prediction strategies at the microplot, field, and regional scales, because these scales differ fundamentally in data requirements, ground-truth sources, output forms, and validation risks.

**Figure 3:**
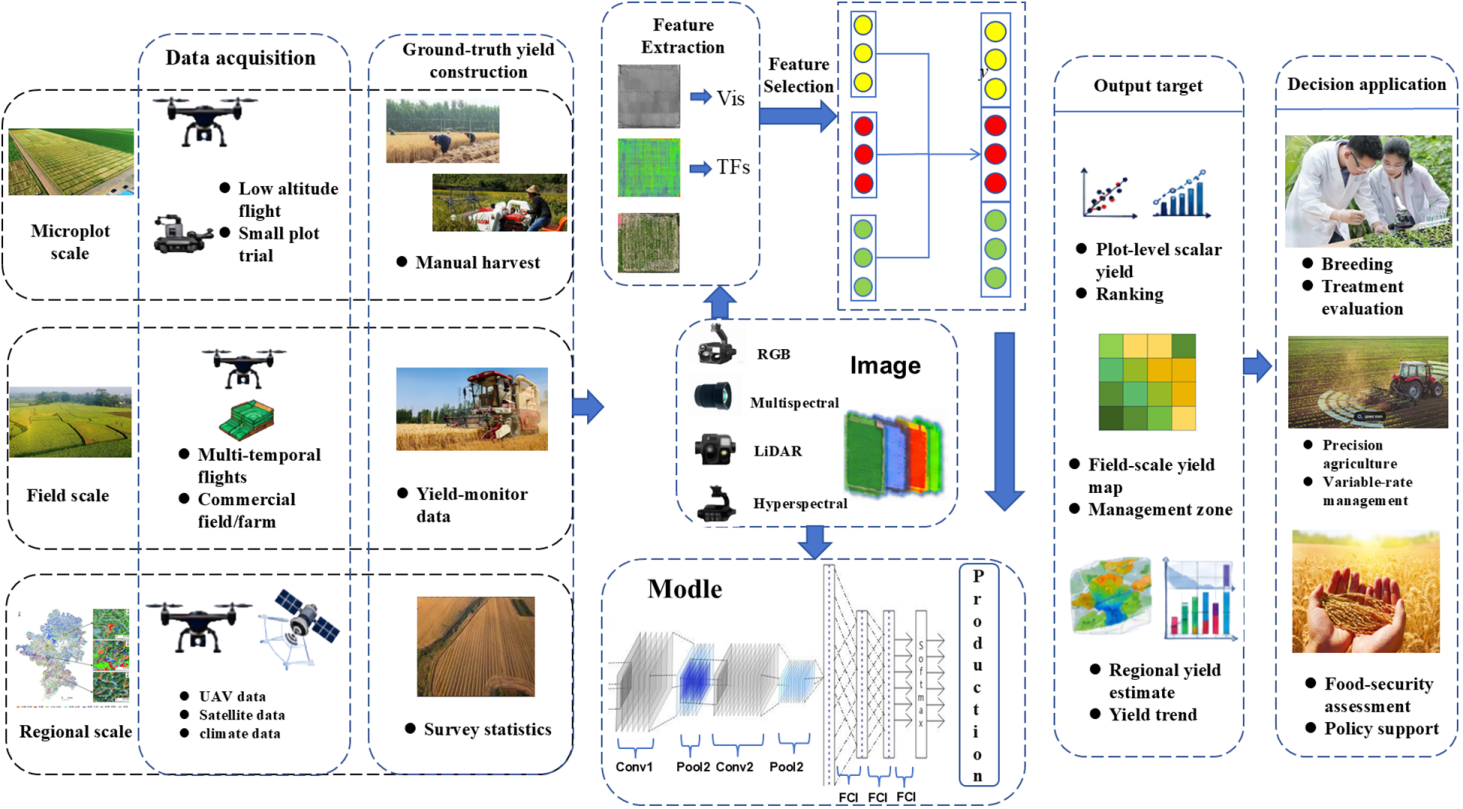
Workflow of UAV-based crop yield prediction at microplot, field/farm, and regional scales. The framework includes data acquisition, ground-truth yield construction, feature extraction and selection, model prediction, output targets, and decision applications corresponding to different spatial scales.

As shown in **Figure 3**, UAV-based crop yield prediction involves a linked workflow from data acquisition and ground-truth yield construction to feature extraction, feature selection, model prediction, output generation, and decision application. At the microplot scale, UAV imagery is mainly used in breeding trials and treatment evaluation, where low-altitude flights provide high-resolution images and yield labels are commonly obtained from manual harvest or plot-combine harvest. At the field/farm scale, UAV-based prediction is more closely related to precision agriculture, where multi-temporal imagery, canopy features, and combine yield-monitor data can be used to generate field-scale yield maps or management zones. At the regional scale, UAV observations are generally used as high-resolution samples and are often integrated with satellite time series, weather/climate data, crop models, and statistical records for regional yield estimation and food-security assessment.

#### 3.1.1 Spatial scale and prediction target definition

A major source of inconsistency in the UAV-based yield-prediction literature is the implicit mixing of spatial scales. At the microplot or quadrat scale, UAV imagery is typically paired with manual harvest or plot-combine measurements, and the model output is commonly a plot-level scalar yield used for breeding evaluation or treatment comparison. At the field or farm scale, UAV imagery is more often used to generate within-field yield maps or management zones, which requires calibration of combine yield-monitor data, moisture correction, coordinate alignment, and filtering of artifacts caused by harvester dynamics (Lyle et al., 2014; A Sudduth et al., 2012). At the regional scale, UAV observations are generally insufficient as a stand-alone data source for large-area yield prediction; instead, they are better used as high-resolution calibration or validation layers in combination with satellite remote sensing, meteorological data, and crop-model frameworks (Peralta et al., 2016; You et al., 2017). Therefore, separating these spatial scales is essential because model inputs, yield labels, validation methods, and practical decision targets are not interchangeable across scales.The differences among microplot, field, and regional prediction scales are summarized in **Table 3** in terms of typical applications, UAV inputs, ground-truth yield sources, output targets, and major risks.

**Table 3:** Spatial scales, ground-truth yield sources, output targets, and major risks in UAV-based crop yield prediction.

Scale	Typical application	UAV/ancillary inputs	Ground-truth yield source	Common output target	Key risk
Microplot	Breeding trials and experimental plots	Centimeter-level RGB, multispectral, hyperspectral, texture, or canopy-height features	Manual quadrat harvest or plot-combine harvest standardized to moisture content	Plot-level scalar yield or genotype/treatment ranking	Small sample size, edge effects, spatial leakage between adjacent plots
Field	Commercial precision agriculture and variable-rate management	UAV mosaics, multi-temporal indices, canopy structure, weather/soil layers	Combine yield-monitor points/maps or whole-field harvested yield	Within-field yield map, management-zone map, or field-average yield	Yield-monitor lag, swath-width and moisture errors, georeferencing mismatch, aggregation bias
Regional	Large production areas, policy, insurance, and food-security forecasting	UAV samples fused with satellite time series, meteorological data, crop models, and statistics	Survey statistics, harvester databases, field records, or calibrated crop-model outputs	Regional yield estimate or gridded yield surface	Upscaling uncertainty, domain shift across years/regions, sparse ground truth

### 3.2 Data acquisition

Data acquisition is a fundamental step in the process of crop yield prediction using UAV remote sensing, playing a decisive role in the accuracy and reliability of predictions. The type of UAV directly affects the spatial scale of data acquisition and operational efficiency, while the type of sensors it carries determines the richness of the data obtained and its potential applications. Therefore, this section will discuss in detail the types of UAV platforms and their accompanying sensor types, aiming to analyze the characteristics and trends of data acquisition methods in current reviews.

#### 3.2.1 Types of UAVs

UAV platforms used for crop yield prediction mainly include multi-rotor and fixed-wing systems (**Table 4**). Multi-rotor UAVs are the dominant choice in plot-based and small-area field experiments. They offer flexible flight, easy operation, low-altitude hovering, and stable image acquisition, which makes them suitable for high-resolution crop monitoring and repeated observations in limited field areas (Boon et al., 2017; Hunt and Daughtry, 2018). In breeding nurseries and precision-agriculture demonstration plots, flights at about 10–100 m can capture detailed canopy spectral, RGB texture, and structural information. Fixed-wing UAVs have longer endurance, higher flight speed, and wider coverage. They are more suitable for rapid monitoring of large fields or contiguous agricultural areas (Liu et al., 2021). These platforms are often operated above 100 m to improve acquisition efficiency (da Silva et al., 2020; Raj et al., 2024). Their stricter take-off and landing requirements and lower maneuverability, however, limit their use in small plots or fragmented fields. Platform selection should therefore match the prediction scale, ground-truth yield acquisition strategy, and application scenario, rather than only reflect a trade-off between spatial resolution and operational efficiency.

**Table 4:** Advantages and disadvantages of multi-rotor UAV vs fixed-wing UAV in the field of agriculture.

UAV Type	Advantages	Disadvantages
Multi-rotor UAVs	- Vertical take-off and landing - Flexible hovering - High resolution - Low cost and easy maintenance - Close-range multi-angle collection	- Poor endurance (20 min -40min) - Only suitable for small-area plots - Not suitable for severe weather - Limited load-carrying capacity
Fixed-wing UAVs	- Relatively long flight range - Strong load-bearing capacity - Relatively fast speed - Strong endurance (1 h- 3 h)	- Restricted take-off and landing - Insufficient flexibility - Not suitable for small-area operations - Relatively high operation difficulty

**Figure 4:**
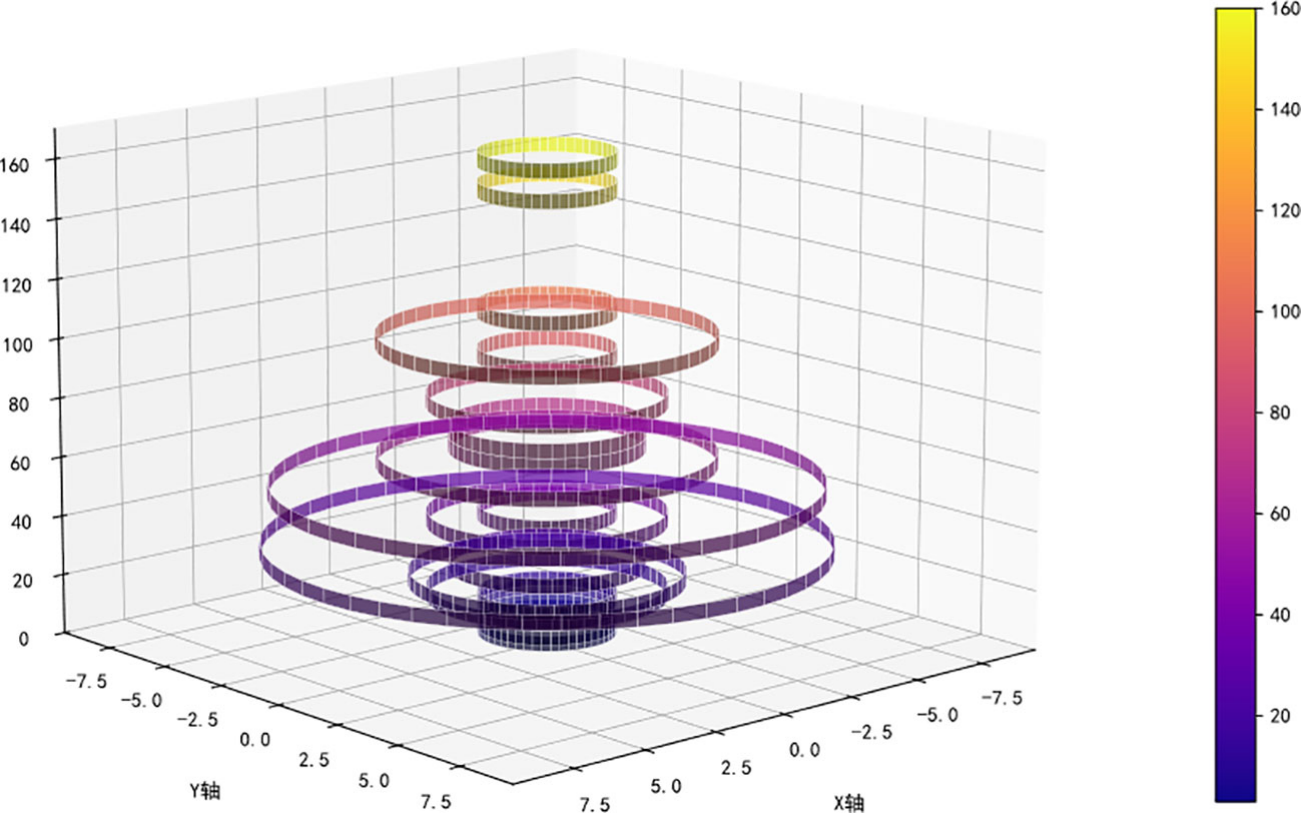
In the literature, there is a statistical chart of UAV flight heights. The area of each ring represents the frequency of flights at that height; the larger the area of the ring, the more frequently this flight height option was utilized.

A comprehensive literature analysis reveals that flight altitude exerts a significant influence on prediction accuracy. We systematically tallied the flight altitude schemes adopted by UAVs in the literature, as illustrated in **Figure 4**, and found that the majority of researchers employ flight altitude strategies ranging from 20 m to 100 m. It is generally believed that higher flight altitudes can improve operational efficiency and reduce flight costs, but they may also decrease image spatial resolution, affecting prediction accuracy. However, empirical studies have shown that this relationship is not simply negative. A study on wild blueberries found that among three flight altitudes—5 m, 15 m, and 30 m—the highest altitude of 30 m actually exhibited the best yield prediction performance. This suggests that flying at greater heights can effectively reduce image noise (such as shadows and soil background factors), highlighting the color and texture differences among different crops, thereby improving the accuracy of crop type identification (Qu et al., 2024). In another study that estimated above-ground biomass of winter wheat using UAV RGB images and texture features, the optimal spatial resolution was found to be 0.5 m, which corresponds to a flight altitude of around 50 m. This height effectively balanced image detail and crop canopy texture information, reducing heterogeneity and background interference within the canopy, thus enhancing estimation accuracy (Yue et al., 2019). Moreover, when there are multiple crop types and complex spatial structures, appropriately lowering the flight altitude to improve spatial resolution can allow for more accurate extraction of crop texture features, thereby improving classification results. However, there is an optimal threshold for this improvement within a certain range; excessively low flight altitudes may lead to reduced accuracy due to excessive detail interference (Böhler et al., 2018). Therefore, in UAV remote sensing for crop yield prediction, selecting the flight altitude requires a comprehensive consideration of crop type, spatial structure, and research objectives. Only by finding the optimal balance point between the efficiency advantage of high-altitude flight and the spatial detail advantage of medium and low-altitude flight can the optimal prediction effect be achieved.

#### 3.2.2 Sensor types

In UAV-based crop yield prediction, sensors play a central role in determining the type, resolution, and reliability of image-derived information (**Table 5**). Current studies mainly employ spectral and RGB sensors, whereas the application of radar and thermal infrared sensors remains relatively scarce.

**Table 5:** Introduction to the sensors carried by the UAV, including the wavelength range, typical resolution, and advantages and limitations.

Sensor type	Wavelength range	Typical resolution	Advantages	Limitations
RGB	- Visible light (Red, Green, Blue)	- High (such as 1–5 cm/px)	- Low cost - Easy to operate	- Limited information, easily affected by lighting
Multispectral	- Visible light - Near - infrared - Red - edge and other additional bands	- Medium (such as 5–10 cm/px)	- Mature processing flow - Rich vegetation information - Can accurately quantitatively evaluate crop health status	- High price, requires precise radiometric calibration
Thermal Infrared	- 8 - 14 μm	- Relatively low(such as 10–20 cm/px)	- Directly reflects thermal characteristics - Conducive to water and stress assessment	- Low resolution - Greatly affected by the environment
Hyperspectral	- Dozens to hundreds ofnarrow bands (including visible light to short - wave infrared)	- Variable, usually low	- Extremely high spectral resolution, can identify microscopic differences in crops	- High equipment cost - Expensive equipment - Large data volume - Complex interpretation
LiDAR	- Near - infrared laser ranging (such as 905 nm or 1550 nm)	- High (point cloud density is adjustable)	- Active ranging, independent of lighting, can obtain high - precision three - dimensional structure	- High cost - Complex operation - Requires professional algorithms to process point cloud data

Multispectral sensors can accurately capture reflectance information of crops across multiple specific spectral bands, including red, green, blue, and near-infrared. Through in-depth analysis of this information, key indices such as the Normalized Difference Vegetation Index (NDVI) and Enhanced Vegetation Index (EVI) can be calculated (Alabi et al., 2022; Mia et al., 2023). Although the formulas for vegetation indices are fixed, variations may arise due to differences in band center and bandwidth; for instance, red light may be at 660 nm or 640 nm, leading to significant discrepancies in the same index. In a study that used the Normalized Difference Water Index (NDWI) to estimate corn canopy water content, researchers employed four different NDWI parameters, resulting in varying experimental outcomes (Chai et al., 2020).

RGB sensors, known for their high accuracy, are mainly used to obtain color and texture information of crop canopies (Herrmann et al., 2020). The color values of leaves of healthy crops are generally within a specific range. When affected by factors such as pests, diseases, or nutrient deficiencies their colors will change significantly. By analyzing characteristics such as color saturation and brightness, it is possible to effectively reflect the growth vitality and environmental stress of crops (Husin et al., 2013; Paranjothi, 2019). Zhang et al. (2021) constructed an RGB model of canopy leaf color deviation distribution pattern, describing canopy color information from three aspects: leaf color depth, distribution bias and concentration for yield prediction. Meina Zhang et al. (2020) were the first to utilize a low-cost UAV RGB imaging system based on ExG color features to predict corn yield. In addition, after converting RGB images to grayscale images, the texture features of the canopy can be analyzed by constructing a grayscale co-occurrence matrix (Alabi et al., 2022; Maimaitijiang et al., 2020; Ren et al., 2023), which can indirectly reflect changes in crop yield.

Hyperspectral sensors, with their excellent spectral resolution, can provide information on up to hundreds or even more continuous spectral bands (Yang et al., 2021), and can extract more vegetation indices compared to multispectral sensors (Berveglieri et al., 2024; Dilmurat et al., 2022; Liu et al., 2023). Hyperspectral bands can also be directly input as feature vectors into the deep learning models to automatically extract the bands sensitive to yield (Fan et al., 2022; Herrmann et al., 2020; Zhang et al., 2019), which is of great value in the information collection for crop yield prediction.

Thermal imaging sensors focus on monitoring canopy temperature, as the degree of water stress and physiological activity in crops directly affect canopy temperature (Elsayed et al., 2017). When crops are short of water, the weakening of transpiration leads to an increase in canopy temperature. The normalized relative canopy temperature (NRCT) data obtained from thermal imaging sensors (Maimaitijiang et al., 2020) can quantitatively assess the water stress status of crops (similar to the Crop Water Stress Index, CWSI). Research indicates that NRCT can effectively characterize the water metabolism efficiency of crops like soybeans through standardized processing combined with canopy temperature extremes (Tmax and Tmin) (Maimaitijiang et al., 2020). Although the predictive accuracy of yield using thermal imaging alone is relatively low, multimodal data fusion has proven that thermal features and spectral saturation features are complementary.Especially in dense canopies, the limitation of spectral information approaching saturation can be broken through. The fusion of thermal features with spectral and structural features significantly improves the model performance (Maimaitijiang et al., 2020; Zhou et al., 2023). It should be noted that canopy temperature can be easily affected by environmental factors such as soil background and atmospheric conditions, requiring radiometric correction and spatial masking techniques (SVM classification to remove soil pixels) to improve data reliability (Maimaitijiang et al., 2020).

LiDAR technology, with its active remote sensing characteristics and three-dimensional structural analysis capabilities, shows significant advantages in crop monitoring. It can penetrate the vegetation canopy to obtain vertical structural features, addressing the limitations of traditional optical remote sensing that only captures two-dimensional spectral information from the canopy surface (Gu et al., 2024). Researchers used LiDAR sensors to achieve centimeter-level precision three-dimensional point cloud reconstructions in wheat and corn fields respectively, and quantified the vertical distribution differences of canopy photosynthetic parameters (P < 0.05). As LiDAR is not constrained by lighting conditions, it can operate in all weather conditions and effectively reduces spectral saturation issues in tall crops like corn, supplementing information about the middle and lower structural layers and significantly enhancing the physical relevance of yield prediction models (Gu et al., 2024; Zhou et al., 2023). However, the application of LiDAR alone still has the problem of missing spectral information, which restricts the accuracy of physiological parameter inversion, along with high equipment costs and complex data processing (Li and Guo, 2015). In response to these issues, the research proposes solutions through multi-source data fusion and algorithm optimization: By combining LiDAR with multispectral/hyperspectral images and using Digital Surface Model (DSM) fusion technology to synchronously extract structural and spectral features, Wenqi Zhou et al. improved the R² of yield prediction to 0.75-0.79 and reduced RRMSE to 1.59%-1.73% in corn studies after fusion.

#### 3.2.3 Ground-truth yield acquisition and label quality

Yield prediction is a supervised-learning task, so reliable yield labels are as important as high-quality UAV imagery. In microplot and breeding-trial studies, ground-truth yield is usually obtained through manual quadrat harvest, whole-plot harvest, or plot-combine harvest. These methods provide direct yield labels, but they are labor-intensive and often produce limited sample sizes (Alabi et al., 2022; Bian et al., 2022; Zhang et al., 2019). Since these labels are commonly aggregated to plot-level yield, UAV-derived features should be extracted within the same plot boundaries. Soil background, shadows, border effects, and non-crop pixels should also be minimized before feature calculation.

At the field and farm scales, combine yield monitors can provide dense georeferenced yield points. These data still require strict quality control before they are used as model labels. Common error sources include grain-flow delay, harvester dynamics at headlands, abrupt changes in travel speed, overlapping harvest passes, inaccurate swath width, variation in grain moisture content, and GNSS positioning errors (Lyle et al., 2014; A Sudduth et al., 2012). UAV-derived features and yield-monitor data should therefore be aligned using a common coordinate system, temporal matching, interpolation or gridding, outlier filtering, and moisture-standardized yield units. When this linkage is weak, a model may show high accuracy under random cross-validation but learn harvest-related artifacts or local spatial clustering rather than transferable crop-yield signals.

### 3.3 Data processing

**Figure 5:**
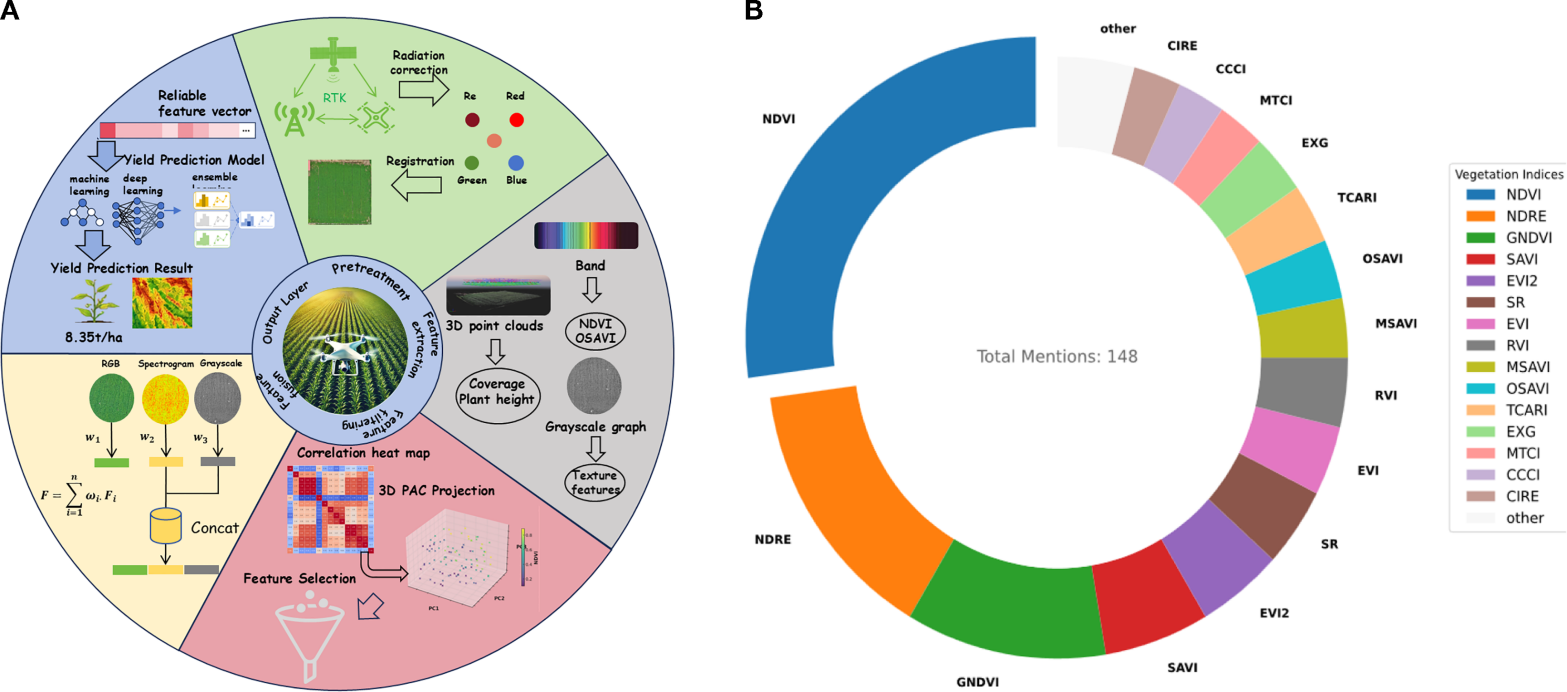
**(A)** General workflow of UAV remote sensing data processing for crop yield prediction. Raw UAV images are first processed through geometric correction, radiometric calibration, image registration, and plot- or field-level clipping. Yield-related features are then extracted, selected, fused, and normalized to construct model-ready datasets for yield prediction. **(B)** Statistical analysis of commonly used vegetation indices in remote sensing crop yield prediction research, totaling 148 references.

The processing of UAV remote sensing data is a crucial step in building precise and reliable crop yield prediction models. Its quality affects not only prediction accuracy, but also the spatial consistency, radiometric comparability, feature stability, and generalization ability of the model. As shown in **Figure 5a**, the general data-processing workflow includes image preprocessing, feature extraction, feature selection and fusion, and the construction of model-ready feature vectors for yield prediction.

After the UAV acquires the original image data, necessary image preprocessing is required, including geometric correction, radiometric correction, and registration operations, etc., to ensure spatial accuracy of the data and the consistency of reflectivity. Geometric correction primarily aims to eliminate spatial position errors caused by the instability of UAV flight posture or lens distortion. The mainstream methods in research rely on ground control points (GCPs) or high-precision RTK positioning systems for geographic registration (Alabi et al., 2022; Herrero-Huerta et al., 2020). Radiometric calibration converts the original digital numbers (DN) into reflectance values using black-and-white calibration panels or built-in sensor algorithms. Especially in multispectral and hyperspectral data, dynamic calibration in combination with a sunlight sensor is required to eliminate the effects of light fluctuations (Alabi et al., 2022; Li et al., 2022; Zhang et al., 2019). Registration is to unify the data obtained from different times, different platforms, and different sensors onto the same spatial coordinate system to ensure the spatial consistency and accuracy of multi-source data (Andrade Júnior et al., 2022; Bhadra et al., 2023; Eugenio et al., 2020).

After completing the image preprocessing, it is necessary to extract feature indicators closely related to crop yield from the remote sensing images, including vegetation indices, texture features, and canopy height. Vegetation indices are typically calculated using multispectral or hyperspectral data, reflecting the growth status of crops (Abderrazak et al., 1996; Myneni et al., 1995; Xue and Su, 2017). The NDVI is widely used in yield prediction due to its simplicity, broad applicability, and stability. Other vegetation indices such as NDRE, GNDVI, and SAVI have also gained attention for their outstanding performance in reducing soil background interference, lowering canopy saturation, and enhancing the sensitivity of vegetation information. Other indices, such as SR, EVI, RVI, MSAVI, OSAVI, TCARI, EXG, MTCI, CCCI, and CIRE, are also used in specific research contexts; their abbreviations, definitions, formulas, and representative references are summarized in **Table 6**. Across the reviewed studies, vegetation indices were mentioned 148 times in total. As shown in **Figure 5b**, NDVI was the most frequently used vegetation index, followed by NDRE, GNDVI, SAVI, EVI2, SR, EVI, RVI, MSAVI, OSAVI, TCARI, EXG, MTCI, CCCI, and CIRE. This distribution indicates that UAV-based yield prediction still relies heavily on conventional greenness and red-edge indices, while soil-adjusted, pigment-sensitive, and color-based indices are used as complementary variables. These indices are sensitive to the physiological characteristics, growth conditions, and stress responses of crops, and are important input variables for yield prediction models (Bian et al., 2022). Canopy structure parameters such as plant height and coverage are often extracted through RGB images, LiDAR, or 3D reconstruction methods. These indicators visually represent the biomass and growth status of crops and make significant contributions to improving the prediction accuracy of the model (Zhou et al., 2021). Additionally, texture features extracted from UAV remote sensing images using methods such as the gray-level co-occurrence matrix (GLCM) are also widely applied in crop yield prediction research. These texture features can capture the spatial heterogeneity of the canopy, significantly enhancing the model’s ability to recognize details of crop growth (Alabi et al., 2022; Maimaitijiang et al., 2020; Ren et al., 2023; Yang et al., 2022).

**Table 6:** Introduction and application of vegetation indices.

Vegetation index	Description	Formula	Reference
NDVI	Normalised difference vegetation index	(NIR - Red)/(NIR + Red)	(Ali et al., 2024; Bao et al., 2023; Berveglieri et al., 2024; Bian et al., 2022; Boon et al., 2017; da Silva et al., 2020; Fei et al., 2022; Fu et al., 2020; Furukawa et al., 2020; Gu et al., 2024; Guo et al., 2022; Herrmann et al., 2020; Hu and Cui, 2023; Hui et al., 2024; Kurihara et al., 2023; Jikai Liu et al., 2022; Luo et al., 2022; Meiyan et al., 2022; Skobalski et al., 2024; Sunoj et al., 2021; Wan et al., 2020; X. Zhang et al., 2019; Zheng et al., 2022; W. Zhou et al., 2023)
NDRE	Normalized difference red edge index	(NIR - Red Edge)/(NIR + Red Edge)	(Ali et al., 2024; Duan et al., 2019; Eugenio et al., 2020; Fu et al., 2020; Hu and Cui, 2023; Hui et al., 2024; Kurihara et al., 2023; Luo et al., 2022; Muruganantham et al., 2022; Qu et al., 2024; Saravia et al., 2022; Shammi et al., 2024; Skobalski et al., 2024; Xiang et al., 2023; B. Yang et al., 2022; Zheng et al., 2022; W. Zhou et al., 2023)
GNDVI	Green normalized difference vegetation index	(NIR - Green)/(NIR + Green)	(Ali et al., 2024; Bao et al., 2023; Duan et al., 2019; Fei et al., 2022; Fu et al., 2020; Hu and Cui, 2023; Hui et al., 2024; Kurihara et al., 2023; Shammi et al., 2024; Skobalski et al., 2024; Sunoj et al., 2021; Xiang et al., 2023; Yang et al., 2022)
EVI-2	Two-band enhanced vegetation index	(2.5 × (NIR − Red))/(NIR + 2.4 × Red + 1)	(Andrade Júnior et al., 2022; Bascon et al., 2022; Luo et al., 2022; Shammi et al., 2024; Sunoj et al., 2021)
SAVI	Soil-adjusted vegetation index	((NIR - Red)/(1 + 0.5))/(NIR + Red + 0.5)	(Andrade Júnior et al., 2022; da Silva et al., 2020; Guo et al., 2022; Luo et al., 2022; W. Zhou et al., 2023)
RVI	Ratio Vegetation Index	NIR/Red	(Bian et al., 2022; Xiang et al., 2023; Zhang et al., 2019)
EXG	Excess green index	2 × Green − Red − Blue	(Skobalski et al., 2024; Sunoj et al., 2021; Zhang et al., 2020)
SCCCI	Simplified canopy chlorophyll content index	NDVI/NDRE	(Barzin et al., 2020; Kumar et al., 2025; Shammi et al., 2024)
GRRI	Green–red ratio vegetation index	Green/Red	(Bian et al., 2022; Shammi et al., 2024; Zeng et al., 2021)
OSAVI	Optimized soil-adjusted vegetation index	1.16(NIR - Red)/(NIR + Red + 0.16)	(Barzin et al., 2020; Eugenio et al., 2020; Hu and Cui, 2023; Zheng et al., 2022)
EVI	Enhanced vegetation index	2.5 × (NIR − Red)/(NIR + 6.0 × Red − 7.5 × Blue + 1)	(Andrade Júnior et al., 2022; Bao et al., 2023; W. Zhou et al., 2023)
WDRVI	Wide Dynamic Range Vegetation Index	(0.05 × NIR - Red)/(0.05 × NIR + Red)	(Eugenio et al., 2020; Luo et al., 2022)
TVI	Transformed Vegetation Index	0.5 × (120 × (NIR - Green)) - 200 × (Red - Green))	(Eugenio et al., 2020; Xiang et al., 2023)
MTCI	Meris Terrestrial Chlorophyll Index	(NIR - Red Edge)/(NIR + Red Edge - Green)	(Bao et al., 2023; Luo et al., 2022; Xiang et al., 2023)
TCARI	Transformed chlorophyll absorption in the reflectance index	3 × [(RE−R) − 0.2(RE−G) × (RE/R)]	(Berveglieri et al., 2024; Kumar et al., 2025)
SR	Simple ratio index	NIR/Red	(Berveglieri et al., 2024; Sunoj et al., 2021)
CIRE	Chlorophyll index red edge	NIR/Red EDGE - 1	(Saravia et al., 2022; Xiang et al., 2023)
CCCI	Canopy chlorophyl content index	(NDRE - NDREmin)/(NDRE_-max_ - NDRE_min_)	(Saravia et al., 2022; Shammi et al., 2024)
LCI	Leaf chlorophyll index	(NIR - Green)/(NIR + Green)	(Gu et al., 2024; Hu and Cui, 2023)

This includes the abbreviations, full names, and calculation formulas of vegetation indices. Here, Red, Green, Blue, NIR, and Red Edge represent the reflectance (digital numbers) measured within their respective wavelength ranges.

Once feature extraction is complete, feature selection and fusion are typically performed to eliminate redundant information and noise, thereby improving the model’s generalization capability (Saravia et al., 2022; Skobalski et al., 2024). With the advancement of data fusion methods, many studies have begun to adopt deep learning or machine learning frameworks to integrate different modalities of remote sensing data, fully tap the potential of multi-source remote sensing information in crop yield prediction (Herrero-Huerta et al., 2020; Mia et al., 2023; Wan et al., 2020; Yang et al., 2024). When inputting feature vectors, to further ensure the generalization ability of the prediction model, data standardization or normalization processing is usually adopted to unify the dimensional differences of feature variables at different scales (Fei et al., 2021; Habibi et al., 2024).

Although these methods share a broadly similar workflow, their specific implementation varies with sensor type, crop species, spatial scale, growth stage, and prediction target. Inadequate preprocessing may introduce radiometric inconsistency, geometric mismatch, feature redundancy, or scale mismatch between UAV-derived variables and yield labels. Therefore, data processing should be regarded as a key factor affecting not only model accuracy, but also the transferability and practical reliability of UAV-based crop yield prediction.

### 3.4 Model construction and input–label–output mapping

**Figure 6:**
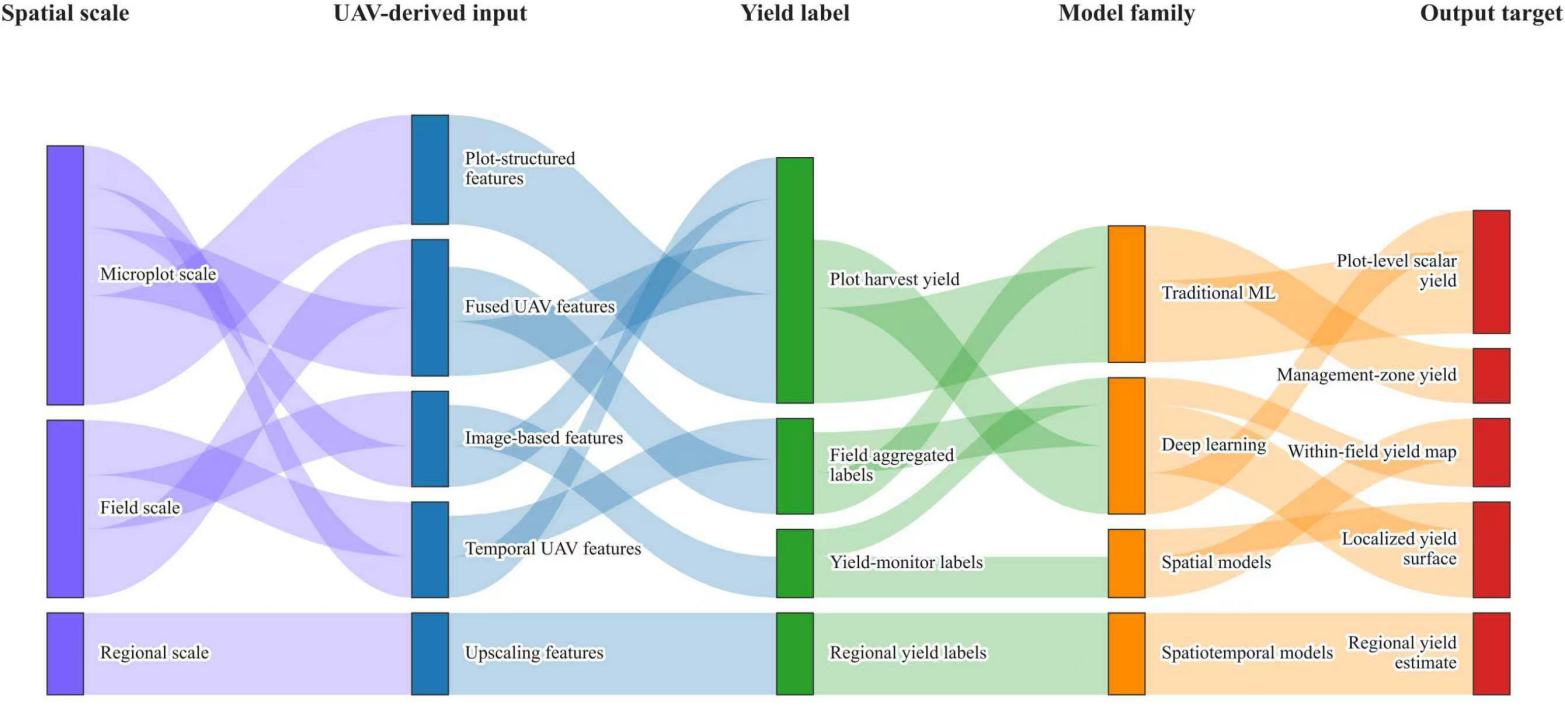
Scale-aware input–label–model–output mapping in UAV-based crop yield prediction studies. The diagram summarizes how spatial scale, UAV-derived input, yield-label source, model family, and output target are connected in the reviewed literature. The link widths indicate dominant mapping patterns summarized from the reviewed studies rather than exact publication counts.

After feature extraction and fusion, model construction determines how UAV-derived variables are linked to ground-truth yield labels and prediction outputs. In the reviewed studies, this relationship is not uniform because different studies use different spatial units, label sources, feature representations, and model families. Plot-level vegetation-index, texture, color, and canopy-structure features are commonly linked with manual harvest, whole-plot harvest, or plot-combine yield labels, whereas field-scale mapping studies require spatially corrected yield-monitor data, and regional-scale studies usually depend on the fusion of UAV observations with satellite, meteorological, soil, crop-model, or statistical data (Zhang et al., 2019; Alabi et al., 2022; Bian et al., 2022; Ren et al., 2023; Lyle et al., 2014; A Sudduth et al., 2012; Peralta et al., 2016; You et al., 2017). Therefore, model performance should not be interpreted only according to algorithm type or reported accuracy metrics. Instead, the spatial support of UAV-derived inputs, the source and processing of yield labels, and the intended output target should be considered together. The conceptual workflow of UAV-based yield prediction is shown in **Figure 6**, and the scale-specific input–label–model–output relationships are further summarized in **Table 7**.

**Table 7:** Input–label–model–output mapping for UAV-based crop yield prediction across spatial scales.

Spatial scale	UAV-derived input	Ground-truth yield label	Model type	Output target	Main risk
Microplot	Plot-level VIs, texture, RGB, canopy features	Manual harvest/plot-combine yield	ML/DL/fusion models	Plot-level scalar yield/ranking	Limited samples; plot-boundary mismatch
Field	Multi-temporal UAV features, canopy maps, image patches	Combine yield-monitor data	ML/CNN/temporal models	Yield map/management zones	Geolocation error; yield-monitor noise
Regional	UAV samples + satellite/weather/soil/crop-model data	Statistics/crop-model outputs	Spatiotemporal models/fusion models	Regional yield estimate/yield trend	Scale mismatch; transferability

As shown in **Figure 6**, UAV-based yield prediction can be organized into three major spatial contexts. At the microplot scale, UAV-derived structured features and fused UAV features are usually extracted within plot boundaries and linked with plot harvest yield labels. These studies mainly produce plot-level scalar yield estimates for breeding trials, genotype ranking, or treatment comparison, and they are commonly implemented using traditional machine-learning models or deep-learning models (Zhang et al., 2019; Alabi et al., 2022; Bian et al., 2022; Ren et al., 2023). At the field scale, fused UAV features, image-based features, and temporal UAV features can support management-zone yield prediction, within-field yield mapping, or localized yield estimation. However, these outputs require yield labels with sufficient spatial consistency and positional accuracy, especially when UAV mosaics are linked with yield-monitor points, interpolated yield surfaces, or management-zone labels (Lyle et al., 2014; A Sudduth et al., 2012; Sunoj et al., 2021; Zhang et al., 2020).

Different model families correspond to different forms of input–output mapping. Traditional machine-learning methods, such as LR, PLSR, RF, SVM, GPR, and XGBoost, are frequently used with plot-level vegetation indices, texture metrics, color indices, canopy-height features, and fused tabular variables to generate plot-level or management-zone yield estimates (Alabi et al., 2022; Fei et al., 2021, Fei et al., 2022; Maimaitijiang et al., 2020; Shammi et al., 2024). Image-based deep-learning models, including CNN, 3D-CNN, attention-CNN, and hybrid CNN models, can learn spatial canopy patterns from RGB, multispectral, or hyperspectral image patches, orthomosaic tiles, and spectral image cubes. However, patch-level or localized predictions are valid only when the corresponding yield labels have sufficient spatial resolution and geolocation accuracy. If a single plot-level yield value is assigned to many image patches, model accuracy may be overestimated because of pseudo-replication or spatial leakage (Zhou et al., 2021; Bhadra et al., 2023; Mia et al., 2023; Tanabe et al., 2023; Zhou et al., 2023).

Temporal UAV features provide another important modeling pathway because they can capture crop-development trajectories and yield-formation processes across growth stages. Multi-date vegetation indices, multi-stage spectral features, temporal image sequences, and phenology-related features are often modeled using LSTM, GRU, CNN-LSTM, CNN-GRU, 3D-CNN, or attention-based temporal networks to predict final yield or stage-sensitive yield responses (Nevavuori et al., 2020; Wan et al., 2020; Tanabe et al., 2023; Wang et al., 2023; Zhou et al., 2023). Nevertheless, the transferability of these models depends on consistent acquisition timing, radiometric calibration, phenological alignment, and cross-year validation. At the regional scale, UAV data are rarely sufficient as a stand-alone data source because of limited spatial coverage. Instead, UAV samples are more commonly used as high-resolution calibration, validation, or scaling information in combination with satellite time series, meteorological data, soil data, crop-model variables, or statistical data to produce regional yield estimates or gridded yield surfaces (Peralta et al., 2016; You et al., 2017).

Overall, the framework and mapping summarized in **Figure 6**, **Table 7** indicate that UAV-based yield prediction should be evaluated as an input–label–output system rather than as a simple comparison of model names. A high R² obtained from plot-level scalar yield prediction should not be directly interpreted as evidence that the same model can generate reliable within-field yield maps or regional yield estimates. Future studies should therefore explicitly report four elements when presenting model performance: the spatial support of UAV-derived inputs, the method used to obtain ground-truth yield, the aggregation or interpolation procedure used to construct model labels, and the intended prediction output. This reporting practice would make accuracy metrics more comparable across studies and reduce the risk of transferring conclusions across incompatible spatial scales.

## 4 Discussion

The literature analysis in Section 3 clarifies the general process of recent research on UAV-based crop yield prediction models, accompanied by visualizations and detailed analyses.During the literature review, a strong emphasis was placed on identifying current challenges and exploring future research directions, which will be discussed in depth in this section.

### 4.1 Methods to improve yield prediction accuracy

#### 4.1.1 Determining the optimal data acquisition period

In research on crop yield prediction based on UAV remote sensing, determining the optimal timing and frequency for data acquisition is a complex and critical issue. High-frequency flights can increase costs and time, while insufficient flights can negatively impact the predictive performance of the models, influenced by the combined effects of crop growth characteristics and environmental factors.

**Figure 7:**
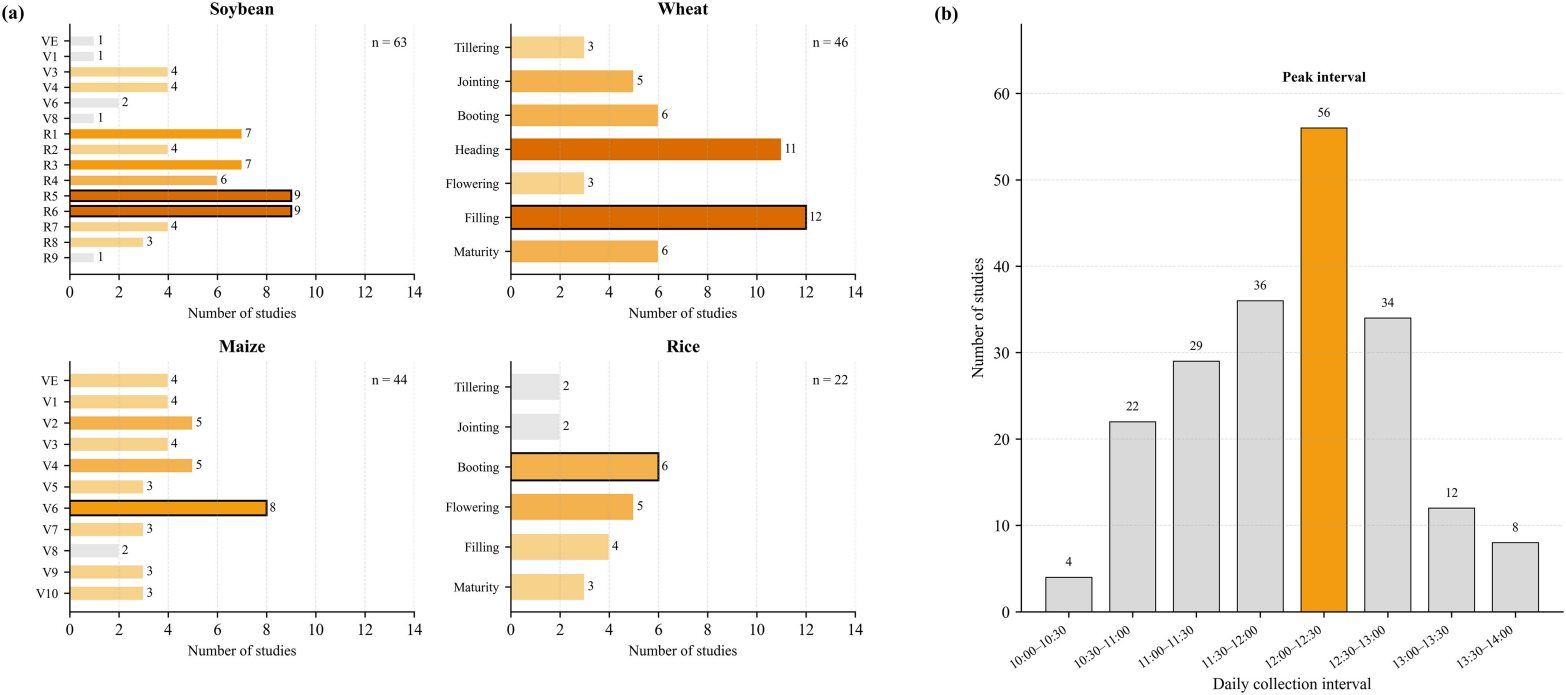
**(a)** In the literature related to remote sensing yield prediction, the frequency distribution of the data acquisition windows for different major grain crops at different growth stages. The four subplots display the occurrence counts of each growth stage for soybeans, wheat, corn, and rice in the studies; the horizontal axis represents the number of collections, while the vertical axis indicates the growth stage codes or names, with the numbers at the top of the bars representing the corresponding frequencies. **(b)** Probability density distribution of UAV remote sensing data acquisition time periods. The histogram uses 10:00–14:00 on the horizontal axis to represent the local times of flights during a day; the vertical axis represents the relative density.

From the perspective of crop growth characteristics, at each stage of the growth cycle of the same crop, various growth indicators change significantly, which is an important factor influencing yield prediction. As shown in **Figure 7**, we have compiled the growth stages at which different crops achieved the highest accuracy rates from the literature. Taking wheat as an example, the heading, flowering, and filling stages are the key periods that determine the final yield. During these reproductive growth stages, the development status of flower organs directly determines its yield rate, and the physiological characteristics and growth conditions of the plants are closely related to the formation of yield. Existing research indicates that multispectral remote sensing images and related indices captured during the heading and flowering stages can effectively reflect the growth trends and yield potential of wheat (Fei et al., 2021; Kang et al., 2024; Tanabe et al., 2023; Tao et al., 2020). Tian et al. (2022) analyzed data of filling, heading, and maturity stages of wheat and found that the yield prediction model established based on the data of filling and heading stages performed quite well (Tian et al., 2022). Different vegetation indices also exhibit certain differences at various times. Liu et al. conducted research and found that the reciprocal ratio vegetation index (repRVI) achieved the best prediction results in the late grain-filling stage, while the MTCI index achieved the best prediction results in the jointing stage (Liu et al., 2022). Furthermore, the booting and flowering stages of rice are crucial for yield formation. The development status of the panicles and the filling degree of the grains during this period significantly affect the final yield. Therefore, the remote sensing data obtained during this stage can better reflect the actual yield potential of the crop. For maize, the V6 stage has been reported as an important observation window, and data collected during this period can improve yield-prediction performance (Kumar et al., 2023). Overall, data acquisition for crop yield prediction should focus on the mid-growth stages of crops, specifically during the rapid growth phase when physiological characteristics are distinctly different, significantly impacting subsequent model inputs.

Environmental factors also play a crucial role in the quality and timing of UAV remote sensing data acquisition (Messina and Modica, 2020). Meteorological conditions such as light, temperature, and humidity can significantly affect model accuracy (Habibi et al., 2024). Through the analysis of relevant research literature (**Figure 7**), it is recommended that ideal data acquisition occurs during clear weather conditions with ample and stable lighting, generally between 10 AM and 2 PM local time. During this period, the solar altitude angle is suitable, and the light is relatively uniform, which can effectively reduce the interference of shadows and light reflection on image quality, and improve the stability and accuracy of remote sensing data. In addition, stable meteorological conditions help to reduce the short-term fluctuations in the physiological state of crops, allowing the data to more accurately reflect the true growth status of the crops. At the same time, during data acquisition adverse weather conditions such as rain and strong winds should be avoided to prevent the surface of the crop canopy from becoming moist, which could cause changes in spectral characteristics and thereby reduce the quality and effectiveness of the remote sensing data.

#### 4.1.2 Feature fusion and selection

Feature selection is a key step in enhancing the generalization ability of models by eliminating redundant variables and noise interference. Due to the numerous vegetation indices that can be extracted from UAV multispectral and hyperspectral data, inputting all vegetation indices into the model without screening may lead to multicollinearity caused by high correlations between different indices (e.g., the NDVI and EVI formulas are similar), resulting in unstable parameter estimates of the model. When the data sample is limited, having too many input variables will increase the risk of overfitting. The general methods for feature selection include calculating Spearman correlation coefficients, LASSO algorithms, multiple linear regression, variance inflation factor (VIF), random forests, and Boruta feature selection, among others (Eugenio et al., 2020; Kumar et al., 2023; Li et al., 2022; Ren et al., 2023; Xiang et al., 2023; Zhang et al., 2019). Zhang et al. determined NDVI and RVI as the optimal combination through a stepwise selection method based on multiple linear regression. Their prediction model achieved a variance of 81.8% for the yield of the soybean plots, proving the sensitivity of the short-wave infrared band to crop water stress and the complementarity of the red light band to chlorophyll absorption (Maimaitijiang et al., 2020). Through the Gini importance assessment of random forest, Xiang Youzhen et al. (2023) selected RVI, NDVI and EVI2 from 14 vegetation indices to form the optimal subset. This resulted in an increase of R² for predicting winter wheat leaf area index to 0.801, indicating that the combination of different spectral bands can effectively distinguish the physiological states of the canopy at different growth stages. Compared with machine learning, the significant advantage of deep learning is the automatic feature extraction (Xiang et al., 2023). Addressing high-dimensional spatiotemporal data, Bhadra et al. compared the feature extraction efficiency of a 3D-CNN architecture, finding that DenseNet automatically captures key patterns of temporal and spatial variation in canopy coverage through dense inter-layer connections. Their model achieved a 23% reduction in complexity after training with 30, 000 samples, and the R² value remained stable at 0.69. This demonstrates the advantage of deep learning in automatically screening out latent features (Bhadra et al., 2023). Furthermore, da Silva et al. (2020) employed path analysis to reveal the causal chain between features and yield: SAVI and NDVI predominantly influence yield variation through leaf area index (direct effect > 0.65), while texture features (such as contrast and entropy) exert indirect effects through adjusting the canopy density. This finding provides a theoretical basis for feature selection based on physiological mechanisms (da Silva et al., 2020).

Due to the limitations of single-sensor data, multi-source data fusion can complement and enhance decision reliability. In the face of complex farmland dynamics, multi-technology collaboration can improve interference resistance. By integrating multi-dimensional features (spectral, texture, temperature), model robustness can be optimized, reducing redundant noise and ultimately achieving precise and stable yield predictions (Killeen et al., 2024). Maimaitijiang et al. (2020) further proposed a multimodal image fusion (MIF) attention mechanism, which dynamically weighting and fusing RGB, hyperspectral near-infrared (HNIR), and thermal infrared image features. The constructed MultimodalNet model achieved adaptive optimization across varieties and irrigation regimes in field-scale predictions of winter wheat, with its dynamic attention allocation strategy significantly enhancing predictive robustness compared to traditional equivalent stacking fusion methods. This systematically validated the synergistic effects of spectral, thermal, and structural features, indicating that the joint analysis of vegetation indices, canopy temperature, and three-dimensional point cloud features can enhance the characterization of photosynthetic efficiency and biomass accumulation (Maimaitijiang et al., 2020). For temporal feature integration, Ren et al. proposed a “growth stage adaptive fusion” method, dynamically weighting vegetation indices (EVI-2, NDVI) and maturity group parameters during different growth periods (from flowering to grain-filling stage) using Gaussian process regression, reducing prediction error (RMSE) by 18.7% compared to static fusion strategies, underscoring the importance of temporal associative features in modeling the yield formation process (Ren et al., 2023). Additionally, Herrero-Huerta et al. (2020)innovatively fused the two-dimensional spectral response of multispectral images with the three-dimensional canopy volume features reconstructed from RGB, utilizing the XGBoost algorithm to analyze the spatial heterogeneity of soybean canopies, achieving an accuracy rate of 91.36%. This confirmed that the coupling of geometric features and spectral features can overcome the limitations of traditional two-dimensional remote sensing analysis.

#### 4.1.3 Innovation and optimization of models

Model innovation and optimization in UAV-based crop yield prediction should be evaluated not only according to reported accuracy, but also according to the correspondence among input data structure, yield-label quality, computational cost, interpretability, and application scale. Ensemble learning has been widely used to improve prediction robustness by combining multiple base learners, which can reduce the overfitting risk of individual models and enhance the stability of prediction results (Fei et al., 2021). The diversity of base learners is an important factor affecting ensemble performance, because heterogeneous learners may capture complementary relationships between UAV-derived features and yield (Saravia et al., 2022). For example, stacking regression has been used to integrate RF, SVM, GP, and RR models, showing improved performance compared with single base learners (Fei et al., 2022). However, increasing the number of base learners does not necessarily guarantee higher accuracy, and model combinations may differ in stability under different data conditions (Fei et al., 2021). The choice of ensemble strategy is also important. Stacking regression, feature-weighted simple fusion, and average ensemble strategies may produce different levels of stability when handling multimodal UAV data (Yang et al., 2024). As UAV remote sensing data become more heterogeneous, ensemble models are increasingly used to integrate multimodal information, but this also increases workflow complexity and may reduce interpretability (Messina and Modica, 2020). Therefore, ensemble models should be assessed not only by accuracy improvement, but also by data dependency, computational cost, and practical applicability when multiple sensor data or multi-period images are fused (Fei et al., 2021, Fei et al., 2022).

**Figure 8:**
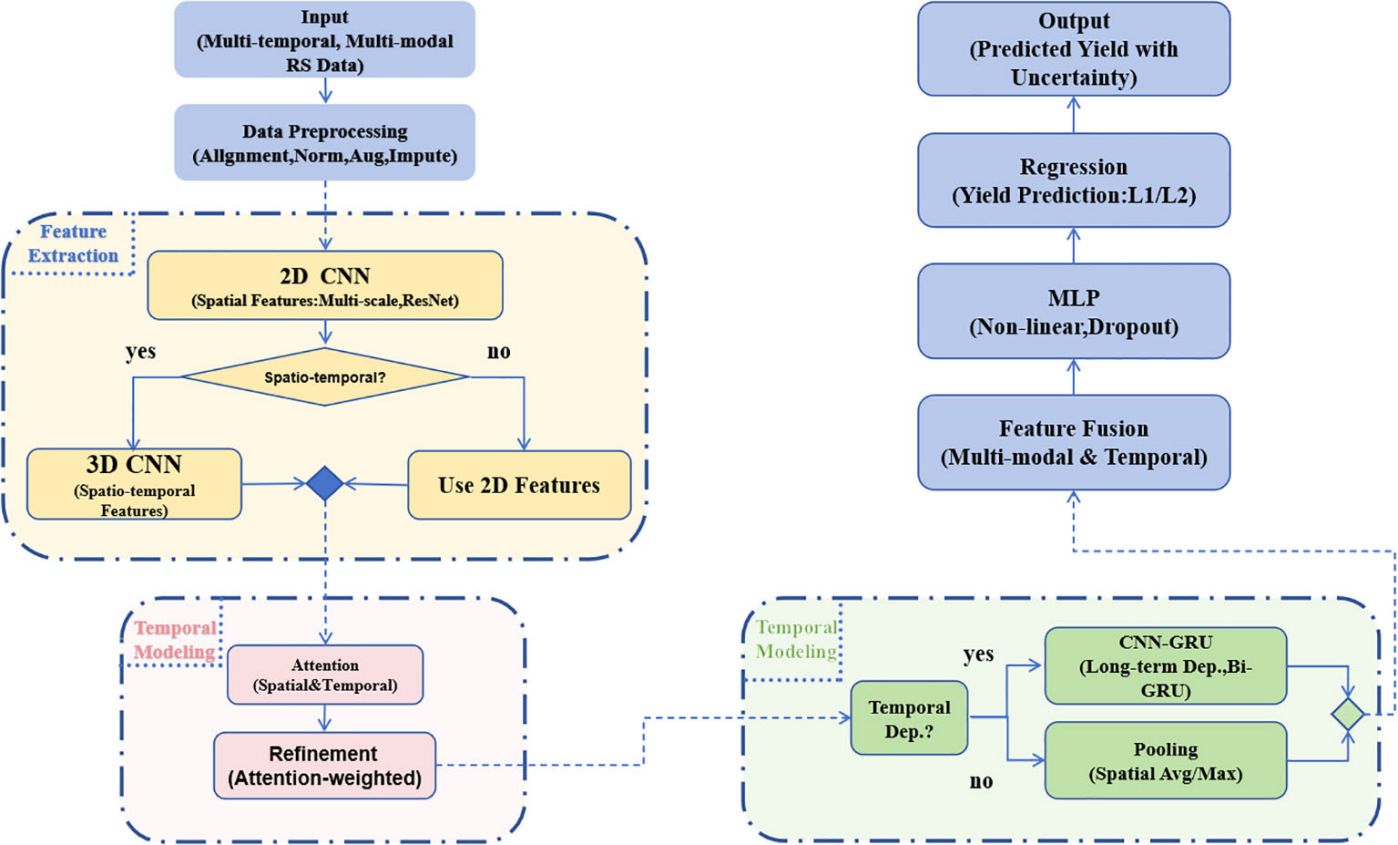
A flowchart of the CNN framework for crop yield prediction, based on existing research. The model utilizes multi-temporal and multi-modal remote sensing data, undergoing preprocessing, spatial and temporal feature extraction, and attention-weighted optimization. Ultimately, it outputs yield prediction results through feature fusion and regression modules, while quantifying prediction uncertainty.

CNN-based and temporal deep-learning models provide another important pathway for UAV-based yield prediction because they can extract spatial, spectral, and temporal patterns directly from image patches, spectral cubes, or multi-date UAV observations. Attention mechanisms can help CNNs emphasize yield-related spatial regions or growth stages within the input data (Zhou et al., 2023). Multi-modal and multi-temporal inputs can further enrich the representation of crop growth conditions, especially when RGB, multispectral, hyperspectral, structural, or meteorological variables are jointly used (Maimaitijiang et al., 2020). Hybrid CNNs and 3D-CNNs extend conventional two-dimensional convolution by incorporating additional spectral, spatial, or temporal dimensions, making them suitable for multidimensional UAV data (Zhou et al., 2021; Bhadra et al., 2023). Similarly, CNN-GRU models combine spatial feature extraction with sequential dependency learning and are therefore suitable for crop growth processes observed across multiple stages (Wang et al., 2023). However, these advantages depend on sufficient labeled samples, spatially reliable yield labels, consistent image acquisition, and adequate computing resources. If plot-level yield labels are repeatedly assigned to many image patches, the reported accuracy may be inflated by pseudo-replication or spatial leakage.The general workflow of CNN-based yield prediction models, including multimodal input, feature extraction, feature fusion, and regression output, is summarized in **Figure 8**.

Therefore, the reviewed model families are compared in **Table 8** in terms of applicable conditions, data dependency, computational cost, and major limitations. This comparison is intended to clarify the practical conditions under which each model family is reliable, rather than to rank models only according to reported R² or RMSE values.

**Table 8:** Cross-comparison of model families used in UAV-based crop yield prediction.

Model family	Applicable condition	Data dependency	Computational cost and main limitation	Representative references
Linear and shallow machine-learning models	Suitable for plot-level prediction using vegetation indices, spectral bands, texture features, or fused tabular variables.	Low to medium; requires extracted UAV-derived features and matched yield labels.	Low to medium cost; limited ability to represent complex spatial or temporal canopy patterns.	(Fei et al., 2021; Fei et al., 2022)
Ensemble learning models	Suitable when multiple base learners or feature groups are combined to improve prediction robustness.	Medium to high; requires multiple model outputs, feature subsets, or fused input variables.	Medium to high cost; may improve accuracy but increase workflow complexity and reduce interpretability.	(Fei et al., 2021; Fei et al., 2022; Li et al., 2022; Yang et al., 2024)
CNN-based models	Suitable for learning spatial canopy patterns from RGB, multispectral, or hyperspectral image patches and orthomosaic tiles.	High; requires image samples with spatially consistent yield labels.	High cost; patch-level prediction may be affected by pseudo-replication or spatial leakage when plot-level labels are reused.	(Mia et al., 2023; J. Zhou et al., 2021; Yamaguchi et al., 2025)
3D-CNN and spectral–spatial CNN models	Suitable for spectral–spatial feature learning from image cubes, multi-band inputs, or interpolated spectral features.	High; requires calibrated spectral images, sufficient samples, and consistent preprocessing.	High cost; sensitive to sample size, image noise, and sensor inconsistency.	(Bhadra et al., 2023; Yamaguchi et al., 2025)
Multi-temporal deep-learning models	Suitable for modeling crop growth trajectories and yield-formation processes from multi-date UAV observations.	High; requires consistent acquisition timing, temporal alignment, and multi-stage UAV features.	High cost; transferability may decrease when acquisition dates, growth stages, or environments differ.	(Tanabe et al., 2023; Wang et al., 2023; Zhou et al., 2023)
Multi-source fusion models	Suitable for integrating canopy spectral, structural, thermal, texture, meteorological, or remotely sensed variables.	High; requires heterogeneous data sources and consistent feature-level or decision-level fusion.	Medium to high cost; fusion can improve robustness but may increase data-processing burden and interpretation difficulty.	(Maimaitijiang et al., 2020; W. Zhou et al., 2023)

Overall, model selection in UAV-based yield prediction should be understood as a trade-off among prediction accuracy, data dependency, computational cost, interpretability, and deployment feasibility. Shallow machine-learning models remain useful when the available dataset is limited and UAV-derived variables have already been extracted as tabular features. Ensemble learning and multi-source fusion can improve robustness by exploiting complementary information from different models or data streams, but they also increase workflow complexity. CNN-based and temporal deep-learning models are more powerful for spatially and temporally structured UAV data, but their reliability depends strongly on label quality, sample independence, acquisition consistency, and computing resources. Therefore, model comparison should move beyond listing model names and reported performance values, and should instead clarify the data and application conditions under which each model family is reliable.

#### 4.1.4 Spatial dependence, scale transfer, and spatiotemporal modeling

Spatial autocorrelation is not only a GIS diagnostic. It is closely related to how model accuracy and generalization should be interpreted. In UAV-based yield prediction, adjacent plots or neighboring field pixels often share similar soil texture, management practices, irrigation status, microclimatic conditions, and phenological timing. When these spatially correlated samples are randomly divided into training and testing sets, the reported R² may be overly optimistic, and the RMSE may be artificially low (Habibi et al., 2024; Killeen et al., 2024). In this case, model performance may reflect local spatial memory rather than transferable crop-yield signals.

Validation design should therefore match the prediction scale. For microplot studies, plot-level or block-level cross-validation can reduce the risk of placing repeated observations from the same plot in both training and testing sets. For field-scale studies, spatially blocked validation and independent-field testing are usually more informative than random partitioning. For regional yield prediction, spatiotemporal modeling is needed because yield patterns are shaped by weather anomalies, soil heterogeneity, management practices, and phenological progression. Conventional tools such as GWR, Moran’s I, and variogram-based diagnostics remain useful. Newer models, including graph-based models, attention-based architectures, 3D-CNNs, CNN-GRU/LSTM networks, and deep Gaussian process models, can further represent spatial neighborhoods and temporal trajectories (Haghighattalab et al., 2017; Nevavuori et al., 2020; Wang et al., 2023; You et al., 2017).

The main implication is that spatial scale, model architecture, and validation design should be reported and interpreted together. A model designed to predict plot-level yield from mean vegetation indices should not be evaluated in the same way as a model designed to generate localized yield maps. Future studies should clearly report the spatial unit of prediction, the spatial unit of validation, and whether the model was tested across plots, fields, years, cultivars, or regions. With this information, spatial autocorrelation can support more credible model evaluation rather than remain a descriptive statistic.

### 4.2 Future research directions: from experimental accuracy to field deployment

Future research on UAV-based crop yield prediction should be guided by deployment bottlenecks rather than by technology labels alone. Key priorities include scale-defined benchmark datasets with rigorous yield-label quality control, field-deployable workflows that integrate UAV, satellite, ground, edge, and cloud data streams, and causal or explainable spatiotemporal modeling frameworks. By considering data scale, label quality, model generalization, and field-operation constraints within a unified framework, UAV-based yield prediction can move beyond experimental accuracy comparison and provide more stable, interpretable, and deployable decision support for agricultural management.

#### 4.2.1 Scale-defined benchmark datasets and ground-truth quality control

Scale-defined benchmark datasets should explicitly document the spatial support of imagery, the source and resolution of yield labels, and the validation-splitting strategy. For microplot studies, this requires standardized plot-boundary extraction, edge-pixel removal, yield moisture correction, and plot-level validation. For field- or farm-scale studies, raw combine yield-monitor data should be systematically cleaned before yield maps are used as model labels. This process should include combine-delay correction, georeferencing assessment, outlier filtering, and uncertainty reporting (Lyle et al., 2014; A Sudduth et al., 2012). Without such quality control, improvements in model architecture may simply compensate for noisy labels rather than reveal robust and transferable crop-yield mechanisms.

#### 4.2.2 Field-deployable UAV-satellite-ground and edge-cloud workflows

Field deployment also requires UAV-based yield prediction to move from isolated UAV flights toward integrated workflows that combine UAV-derived fine-scale information, satellite-based temporal continuity, ground observations, and farm-machinery data. UAV imagery is well suited for high-resolution calibration and local diagnosis, but regional or season-long yield prediction usually requires satellite time series, meteorological data, soil information, and crop-management records to provide temporal continuity and broader spatial coverage. In this context, edge–cloud workflows should be evaluated not only by inference speed, but also by their ability to operate under real field constraints, including onboard power supply, payload capacity, communication bandwidth, image tiling, model compression, illumination and wind variability, data synchronization, and compatibility with farm operations. Similar deployment constraints have also been emphasized in recent UAV-based field-crop-counting studies, where semantic gaps, temporal decay, economic feasibility, and workflow integration were identified as key barriers to transferring deep-learning models from controlled datasets to operational field applications (Pu et al., 2025). For yield prediction, these issues suggest that crop counting, canopy-structure characterization, UAV–satellite data fusion, and yield estimation should be connected as a unified decision chain rather than treated as separate computer-vision or remote-sensing tasks.

#### 4.2.3 Causal and explainable spatiotemporal modeling for decision-level trust

Causal interpretability is important for making UAV-based crop yield prediction reliable in practical applications. Methods such as SHAP, LIME, attention heatmaps, and partial-dependence plots can identify influential variables. However, these *post hoc* explanations alone cannot prove that a model has learned agronomically meaningful mechanisms. A more reliable approach is to combine explainable artificial intelligence with crop physiology, phenology, and management knowledge. Spectral indices should be interpreted in relation to chlorophyll content, biomass accumulation, water stress, canopy closure, and grain filling. Texture features and LiDAR-derived variables should be linked to canopy architecture, stand uniformity, and plant spatial distribution. Temporal features should be connected to key stages of yield formation. With these agronomic and physiological constraints, models can reduce their dependence on spurious correlations caused by soil background, illumination variability, management zones, or harvest-map artifacts. This can improve trust among growers, breeders, and agricultural decision makers.

## 5 Conclusion

This review synthesized 70 peer-reviewed studies published between 2018 and 2025 on UAV-based yield prediction for soybean, corn, wheat, and rice. Using a “Data–Ground Truth–Model–Decision” framework, it examined how UAV platforms, sensor configurations, feature engineering, yield-label acquisition, model construction, validation design, and decision outputs jointly shape prediction reliability. The reviewed studies show that UAV remote sensing has strong potential for high-resolution crop yield prediction. Multispectral, RGB, hyperspectral, thermal, LiDAR, and fused data can capture crop growth, canopy structure, and stress-related information at different growth stages. Machine-learning and deep-learning models further improve the use of these data for yield estimation.

The main limitation is that high prediction accuracy does not always indicate strong practical reliability. UAV-derived features, ground-truth yield labels, and model outputs must be aligned at the same spatial scale. A plot-level yield model, a field-scale yield map, and a regional yield estimate require different label sources, validation strategies, and decision targets. Random validation can also overestimate model performance when spatially related samples or repeated observations are shared between training and testing sets. For this reason, future studies should report prediction scale, yield-label resolution, validation design, and transfer-testing conditions more clearly.

UAV-based crop yield prediction is moving from experimental accuracy comparison toward field-oriented decision support. Further progress will depend on scale-defined benchmark datasets, rigorous ground-truth yield quality control, UAV–satellite–ground data fusion, spatially aware validation, causal and explainable spatiotemporal modeling, and edge–cloud workflows that can operate under real field conditions. By integrating data scale, label quality, model generalization, and deployment constraints, UAV-based yield prediction can become more robust, interpretable, and useful for breeding evaluation, precision field management, and agricultural decision making.

## Data Availability

The original contributions presented in the study are included in the article/supplementary material. Further inquiries can be directed to the corresponding authors.
